# Polycythemia with Renal Cell Carcinoma and Normal Erythropoietin Level

**DOI:** 10.1155/2019/3792514

**Published:** 2019-12-11

**Authors:** Jonathan Kopel, Pranav Sharma, Irfan Warriach, Sriman Swarup

**Affiliations:** ^1^Texas Tech University Health Sciences Center, School of Medicine, Lubbock, TX, USA; ^2^Department of Urology, Texas Tech University Health Sciences Center, Lubbock, TX, USA; ^3^Department of Pathology, Texas Tech University Health Sciences Center, Lubbock, TX, USA; ^4^Department of Hematology and Oncology, Texas Tech University Health Sciences Center, Lubbock, TX, USA

## Abstract

A 61-year-old obese Caucasian male with past medical history of smoking, hypertension, chronic obstructive pulmonary disease (COPD), and sleep apnea presented to the hematology clinic with polycythemia. Despite the newly-diagnosed polycythemia, the patient denied any significant symptoms or history of blood clots. Further evaluation with computerized tomography (CT) and ultrasound showed a large renal mass suspicious for renal cell carcinoma of the right kidney. An incidental abdominal aortic aneurysm (AAA) measuring was also appreciated on imaging. Subsequent histological sections of the tumor showed cell renal cell carcinoma. Though previously reported, the concomitant finding of an AAA with renal cell carcinoma with a normal erythropoietin levels is surprising. Given the surgical complications associated with concomitant conditions with renal cell carcinoma, further investigation into paraneoplastic syndromes secondary to renal cell carcinoma remains open to investigation.

## 1. Introduction

Despite being the 14^th^ most common cause of cancer, renal cell carcinoma accounts for 85% of all renal neoplasms [1]. Although the cancer classically presents with a palpable mass, hematuria, and flank pain, over 10−40% of renal cell carcinoma patients exhibit paraneoplastic syndromes, particularly hypercalcemia, hypertension, and polycythemia [2–4]. As many urologists concede, renal cell carcinomas are medicine's “great masqueraders” affecting multiple organs from a primary cancer [4]. Current studies suggest that unregulated secretion of signaling molecules and antigen mimicking from tumor cells are the primary culprits producing organ damage associated with paraneoplastic syndrome [4, 5]. Paraneoplastic syndromes also influence the severity and metastasis of primary tumors [4]. For example, patients with hypercalcemia secondary to renal cell carcinoma are associated with higher-stage lesions and bone metastasis while those with hypertension acquire low-grade tumors of clear-cell histology [4]. A rising number of care reports describe several unique paraneoplastic syndromes associated with renal cell carcinoma ranging, including intractable coughs, limbic encephalitis, and bullous pemphigoid [6–8]. In general, patients with concurrent renal cell carcinoma and paraneoplastic syndromes have worse outcomes after undergoing nephrectomy compared with incidentally found tumors. Furthermore, the mechanisms underlying the synergistic actions of renal cell carcinomas and paraneoplastic syndromes remains elusive [9]. Therefore, renal cell carcinomas provide a unique opportunity to investigate the mechanisms and clinical manifestations cancer has upon organ system function and integrity. In this case report, we report polycythemia secondary to renal cell carcinoma with normal erythropoietin (EPO) and an incidental abdominal aortic aneurysm (AAA). Specifically, the finding of normal EPO levels in the presence of a paraneoplastic syndrome is both surprising and unique given the large amount of literature demonstrating elevated EPO levels with renal cell carcinoma [10]. In the following case report, we hope to contribute to the diversity and complexity associated with concomitant conditions secondary to renal cell carcinoma while providing a plausible mechanism of action.

## 2. Case Report

A 61-year-old obese Caucasian male with past medical history of smoking, hypertension, chronic obstructive pulmonary disease (COPD), and sleep apnea presented in consultation to the hematology clinic with newly-diagnosed polycythemia with an elevated hemoglobin level (22.4 g/dL), elevated hematocrit (66.9%), and high partial thromboplastic time (40.9 sec). The polycythemia with high hemoglobin and hematocrit levels were first discovered during a preoperative work-up for a planned elective rotator cuff surgery. Due to concern for increased clot formation during elective surgery, the patient was referred to the hematology clinic for further evaluation and treatment.

**Figure 1:**
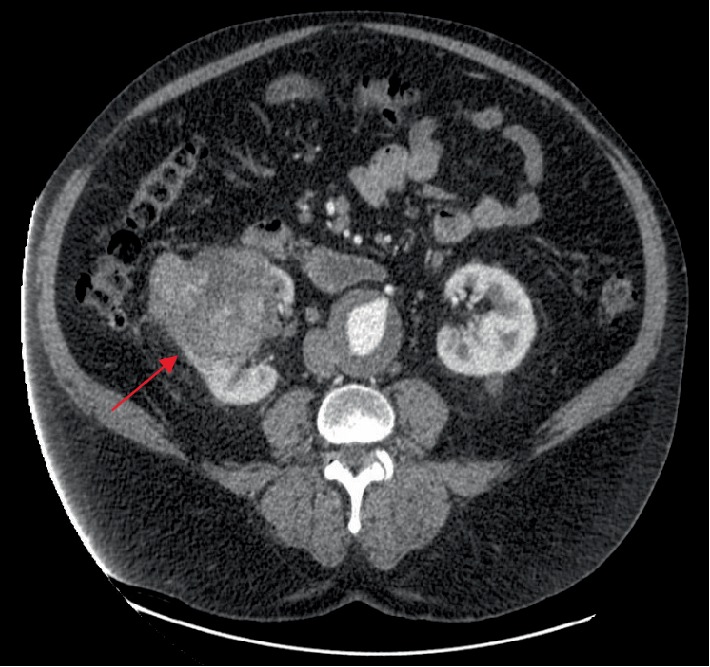
Right lower pole, anterior renal mass.

**Figure 2:**
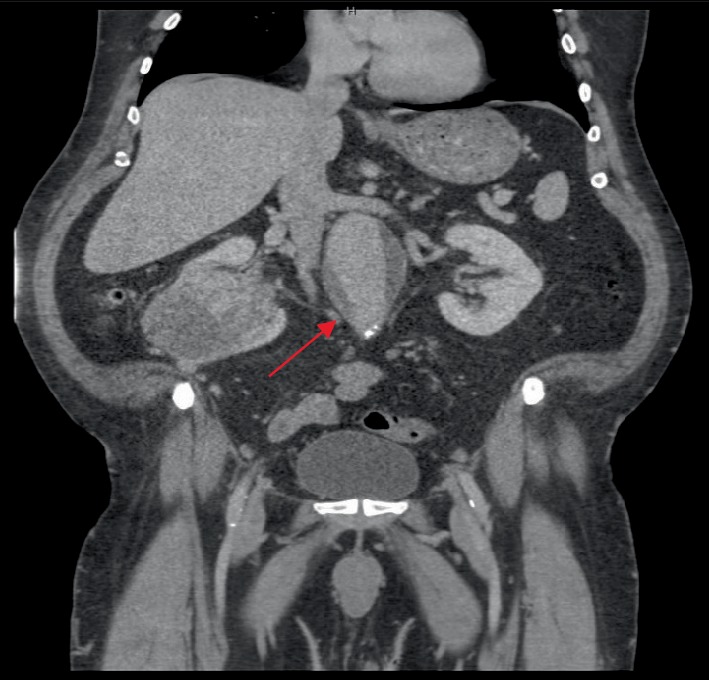
Abdominal aortic aneurysm (AAA).

Despite the newly-diagnosed polycythemia, the patient denied any significant symptoms or history of blood clots. Further evaluation by the hematologist was conducted using a renal ultrasound as well as cross-sectional imaging of the abdomen and pelvis with computerized tomography (CT) scan to determine the underlying cause of the polycythemia in this patient. The ultrasound and CT scan imaging showed a large 10 cm right lower pole, anterior renal mass suspicious for renal cell carcinoma of the right kidney, as shown in Figure 1. The patient's renal cell carcinoma was diagnosed as a T2N0M0 according to the Tumor, Nodes, and Metastasis staging system, which evaluates a renal cell carcinoma according to its size/infiltration to surrounding anatomical structures, lymph node enlargement, or metastasis to surrounding organs [11]. A paraneoplastic syndrome was suspected to account for the patient's polycythemia and elevated hemoglobin. An incidental AAA measuring 6 cm in the juxtarenal position with normal bilateral iliac arteries was also appreciated on imaging, as shown in Figure 2.

For definitive diagnosis and treatment of the patient's suspected right lower pole, anterior renal mass as well as treatment of the patient's polycythemia secondary to a suspected paraneoplastic syndrome, open right radical nephrectomy was recommended. Additionally, given the current size of the juxtarenal AAA with >50% risk of rupture, open repair of the AAA with Dacron tube graft was also recommended in the same operative setting.

**Figure 3:**
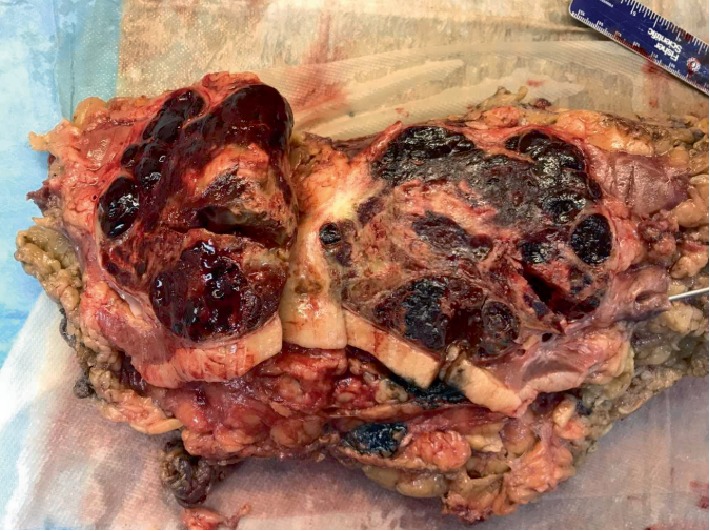
Renal carcinoma confined to capsule.

**Figure 4:**
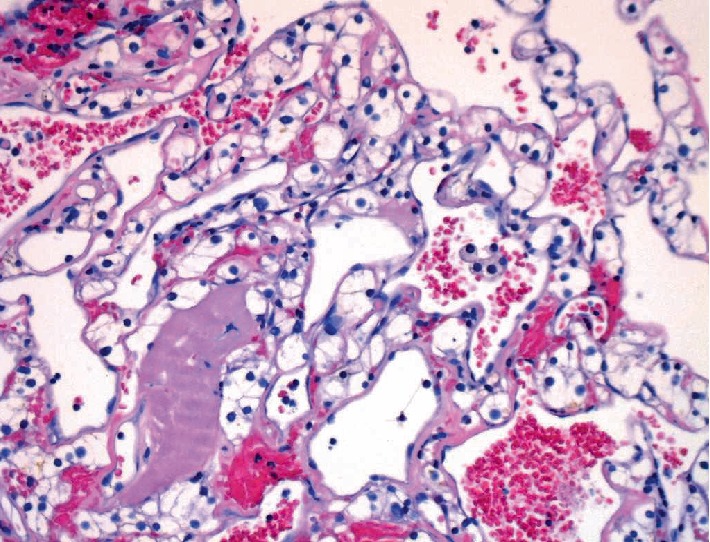
Microscopy image of resected tumor showing clear cell renal cell carcinoma.

Subsequently, an open repair of the juxtarenal AAA with a 24 mm Dacron tube graft with open right radical nephrectomy was successfully performed on the patient several days later through a large, midline abdominal incision with minimal blood loss (50 ml). There were no immediate perioperative or postoperative complications, and the patient recovered well after the surgery and was discharged home on postoperative day 5. The patient's preoperative hemoglobin and erythropoietin level was 18.3 g/dL and 8.5 IU/L respectively, which normalized to a hemoglobin level of 13.1 g/dL within 2-3 days after the surgery. Subsequent measurements months after the surgery measured hemoglobin to be 12.0 g/dL. The patient's blood pressure pre- and postoperatively was measured to be 145/85 mmHg to consistently 120/70 mmHg. No further treatment or surgical interventions were required. The tumor was confined by renal capsule, limited to the kidney situated 1 mm away from the renal pelvis, as shown in Figure 3. Histological sections of the tumor showed predominantly hemorrhagic and infarcted tumor with only a small focus of viable tumor. The sections showed tumor cells with abundant clear cytoplasm and inconspicuous nucleoli consistent with a clear cell renal cell carcinoma Fuhrman grade 1, as shown in Figure 4. The patient was followed for a year and half after the surgery with significant improvement on physical exam and normalized hemoglobin, blood pressure, and EPO values.

## 3. Discussion

In this case report, the patient exhibited two of the three most common paraneoplastic syndromes associated with renal cell carcinoma: polycythemia and hypertension. Although several mechanisms have been proposed to explain hypertension in renal cell carcinoma patients, current studies suggest that the vasoactive peptides endothelin-1, urotensin-II, and adrenomedullin are likely to be involved [12–18]. Furthermore, polycythemia also contributes to hypertension through the upregulation of hypoxia inducible factor-1 (HIF-1) from renal cancer and epithelial cells increasing EPO production [19–21]. Under physiological states, tissue hypoxia stimulates peritubular fibroblast in the renal cortical labyrinth producing EPO, which stimulates the bone marrow to produce red blood cells [22]. In many renal cell carcinomas, EPO receptors in the renal proximal tubule are overexpressed hastening tumor growth through increased angiogenesis [22]. Furthermore, increased EPO receptor expression is correlated with a worse prognosis in renal carcinoma patients [23, 24]. Despite elevated EPO receptor and serum levels, a significant proportion of renal cell carcinoma patients exhibit anemia and polycythemia, which may result from EPO hyporesponsiveness, iron deficiency, and chronic inflammation [10].

Among renal cell carcinoma patients, 66% have elevated EPO levels despite only 8% having polycythemia [4]. Furthermore, 20% of renal cell carcinoma patients have hypercalcemia while 40% have hypertension [4]. In this light, the finding of renal cell carcinoma and normal EPO levels was both surprising and unique. In this patient, a significant proportion of the renal cell carcinoma was necrotic upon examination, which possibly decreased the number of EPO secreting cells and lowered serum EPO levels. The normal EPO level is likely to involve in the renal cell carcinoma, contralateral kidney, and the patient's medical comorbidities. One study suggests that renal cell carcinoma cells may produce an inactive form of EPO, which may explain the lack of erythrocytosis in renal cell carcinoma patients with elevated EPO levels [25]. However, the contralateral kidney of the patient was unaffected and had the ability to compensate for the loss of EPO. A retrospective analysis of partial or complete nephrectomies showed that the contralateral kidney successfully maintained renal function and hemodynamics for several years after surgical resection [26]. Therefore, it is likely the contralateral kidney may compensate for reduced EPO production and other hemodynamic factors necessary for maintaining proper homeostasis. A recent study also found male gender and tumor size were positively correlated with an increase in contralateral kidney volume after a complete nephrectomy [27]. Therefore, the complete nephrectomy puts an increases metabolic requirement for the contralateral kidney to maintain the proper homeostasis through secretion of EPO and other humoral factors.

Furthermore, the patient's concurrent COPD, sleep apnea, and smoking history should increase EPO levels through hypoxic stimulation. Measurements of EPO levels in COPD found higher levels of EPO levels under anemic conditions [28, 29]. However, chronic COPD exacerbations can produce low EPO levels through systemic inflammatory processes mediated by proinflammatory cytokines, such as interleukin-1 (IL-1) and tumor necrosis factor-*α* (TNF-*α*) [29–31]. Sleep apnea can stimulate EPO production in the contralateral side through hypoxic stimulation. Interestingly, sleep apnea is associated with increased renal cell carcinoma tumor growth through vascularization processes mediated by vascular endothelial growth factor (VEGF) [32]. The patient's sleep apnea in this case may explain the significant tumor growth and necrotic tissue present in the gross pathology examination. Although no study has systematically examined the mechanism underlying renal cell carcinoma and low EPO levels, the patient's history of COPD, smoking, and sleep apnea and the presence of a functioning contralateral kidney provide a mechanism through which EPO levels are maintained during and after the removal of a renal cells carcinoma.

Further investigation is required to elucidate the mechanism underlying a decrease in EPO level in the presence of both renal cell carcinoma and chronic respiratory syndromes. In either case, surgical removal of the renal cell carcinoma and repair of surrounding vasculature successfully resolve most complications [33–35]. Specifically, it is unknown whether serum EPO levels correlate with the severity of renal cell carcinoma evaluated through the TNM. Given the rising interest and reports of surgical complications of concomitant conditions with renal cell carcinoma, more investigation into the mechanism, occurrence, and additional risk factors posed by these paraneoplastic syndromes secondary to renal cell carcinoma remains open to investigation [34, 36, 37].
